# Post-introduction evolution in the biological control agent *Longitarsus jacobaeae* (Coleoptera: Chrysomelidae)

**DOI:** 10.1111/j.1752-4571.2012.00264.x

**Published:** 2012-12

**Authors:** Marianna Szűcs, Urs Schaffner, William J Price, Mark Schwarzländer

**Affiliations:** 1 Department of Plant, Soil, and Entomological Sciences, University of IdahoMoscow, ID, USA; 2 Department of Bioagricultural Sciences and Pest Management, Colorado State UniversityFort Collins, CO, USA; 3 CABI Europe – SwitzerlandDelémont, Switzerland; 4 Statistical Programs, University of IdahoMoscow, ID, USA

**Keywords:** aestivation, biological control agent, body size, climatic adaptation, contemporary evolution, larval development

## Abstract

Rapid evolution has rarely been assessed in biological control systems despite the similarity with biological invasions, which are widely used as model systems. We assessed post-introduction climatic adaptation in a population of *Longitarsus jacobaeae*, a biological control agent of *Jacobaea vulgaris*, which originated from a low-elevation site in Italy and was introduced in the USA to a high-elevation site (Mt. Hood, Oregon) in the early 1980s. Life-history characteristics of beetle populations from Mt. Hood, from two low-elevation sites in Oregon (Italian origin) and from a high-elevation site from Switzerland were compared in common gardens. The performance of low- and high-elevation populations at a low- and a high-elevation site was evaluated using reciprocal transplants. The results revealed significant changes in aestival diapause and shifts in phenology in the Mt. Hood population, compared with the low-elevation populations. We found increased performance of the Mt. Hood population in its home environment compared with the low-elevation populations that it originated from. The results indicate that the beetles at Mt. Hood have adapted to the cooler conditions by life-history changes that conform to predictions based on theory and the phenology of the cold-adapted Swiss beetles.

## Introduction

Adaptive phenotypic changes in contemporary time scales have been reported in several species in response to rapid change in climatic regimes (reviews in Hendry and Kinnison 2001; Kinnison and Hendry 2001; Reznick and Ghalambor 2001; Parmesan and Yohe 2003; Root et al. 2003; Stockwell et al. 2003; Parmesan 2006). In many instances, the observed life-history, morphological, behavioral, or physiological changes are due to phenotypic plasticity of the organisms (Gienapp et al. 2008). However, evolutionary, that is, genetically based changes have also been documented, and in some cases, these changes occurred over decades or a few hundred years (Reznick and Ghalambor 2001; Stockwell et al. 2003). Biological invasions have provided great model systems as many reported cases of contemporary evolution are associated with colonization events (Reznick and Ghalambor 2001; Lee 2002; Stockwell et al. 2003; Cox 2004; Lambrinos 2004; Bossdorf et al. 2005; Whitney and Gabler 2008).

Biological control introductions share many of the same characteristics as invading species that can theoretically promote rapid evolution, yet post-introduction evolution of biological control agents is largely unexplored (Roderick and Navajas 2003; Hufbauer and Roderick 2005; Phillips et al. 2008). This is surprising, given the number of introductions and subsequent establishments of classical biological control agents in distinct environments over the past 120 years and the fact that both the geographic source(s) of initial releases and the founding population sizes are often well documented (Julien and Griffiths 1998). One of few studies to explicitly test evolution in a biological control agent found that maladaptive evolution has occurred in a parasitoid (*Aphidius ervi* Haliday) released to control pea aphids (*Acyrthosiphon pisum* Harris) (Hufbauer 2002). Phillips et al. (2008) reported adaptive evolution in the asexually reproducing parasitoid *Microctonus hyperodae* (Loan), where selection has favored one of two biotypes released in New Zealand. With so little work done on contemporary evolution in biological control agents, its prevalence is unknown. However, as biological control agents introduced into a new environment are likely to experience novel selection pressures, contemporary evolution may play an important role in the establishment, population growth, and spread of introduced biological control agents. Hence, the prevalence of contemporary evolution in biological control agents and its potential influence on the outcome of classical biological control projects should be assessed more thoroughly.

Here, we evaluated evidence for post-introduction adaptive evolution in the ragwort flea beetle, *Longitarsus jacobaeae* Waterhouse. *Longitarsus jacobaeae* was introduced to the United States about 40 years ago from Italy for the biological control of tansy ragwort, *Jacobaea vulgaris* Gaertn. These beetles have since successfully established in a range of environments, including high-elevation habitats with a winter-cold continental climate and significantly shorter growing seasons relative to their Mediterranean source region (McEvoy et al. 1991; Littlefield et al. 2008; Szűcs et al. 2011). We set out to test whether US populations at high elevation have undergone rapid adaptive evolution enabling them to establish and thrive in this new environment. In accordance with life-history theory (Abrams et al. 1996; Roff 2002) and empirical data (Orr 1996; Berner et al. 2004), we predicted a shift in phenology either due to accelerated development, which could lead to smaller adult size or earlier onset of larval development in response to the shorter growing seasons. Moreover, we expected changes in photoperiod controlled traits, such as adult aestival diapause, that are most likely to respond quickly to altered climatic conditions (Bradshaw and Holzapfel 2008). The predictions were tested using four distinct collections of beetles: a high-elevation US population (Mt. Hood, Oregon), two low-elevation US populations (Salem, Oregon which served as the source for the Mt. Hood releases; and Scherzinger, Oregon which served as a replication of a low-elevation population; Kawecki and Ebert 2004), and one high-elevation population from the Swiss Jura Mountains. We expected that Salem and Scherzinger beetles would do poorly in the high-elevation environment and that the phenology of the Mt. Hood population would more closely resemble that of the cold-adapted Swiss population than that of the low-elevation Oregon populations (Italian ancestry) from which the Mt. Hood population originated. To test our hypotheses, we compared life-history traits and the phenology of populations in laboratory and field experiments and conducted a partial reciprocal transplant of beetles between a low- and high-elevation site.

## Materials and methods

### Introduction history and biology of *Longitarsus jacobaeae*

The univoltine *L. jacobaeae* (Coleoptera, Chrysomelidae) was introduced from a low-elevation site near Rome, Italy, to Fort Bragg, California, in 1969 to control the invasive tansy ragwort (*J. vulgaris* Gaert. = *Senecio jacobaea* L.) (Frick 1970). Beetles established in California were redistributed to coastal areas of Oregon from the early 1970s (Isaacson 1978). Beetles collected at lowland Salem, Oregon, between 1978 and the early 1980s were used to found a population at Mt. Hood, Oregon, the focal population of this study (Hawkes 1980; E. Coombs personal communication). A cold-adapted strain of *L. jacobaeae* was introduced from the Swiss Jura Mountains, near St. Imier (933-m elevation, 47°09′N, 6°59′E) and Mettembert (701-m elevation, 47°24′N, 7°20′E), to Montana in 2002 (Littlefield et al. 2008).

**Figure 1:**
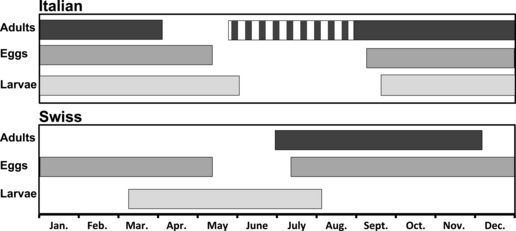
Comparison of *Longitarsus jacobaeae* life cycles from Italy and from Switzerland (Frick and Johnson 1973; Puliafico et al. 2008). Dashed column indicates the characteristic aestival (reproductive) diapause of Italian beetles during the hot and dry summer. Note that egg, larval, and adult stages may all be present during the winter in Italy, and the marked differences in egg diapause, whereas larvae only emerge in the spring in winter-cold Switzerland.

The phenology of beetles in the native Eurasian range varies widely in different climates, as evidenced from life-cycle descriptions of populations from England, the Netherlands, Switzerland, and Italy (reviewed by Windig 1991). Here, we focus on Italian beetles, as the source of the initial biological control effort and Swiss beetles from high elevation as a point of comparison. These populations show marked differences in egg and adult diapause characteristics and in emergence time of larvae and adults (Frick 1971; Frick and Johnson 1972, 1973; Puliafico et al. 2008) (Fig. 1). Italian beetles emerge in late spring and after a brief feeding period on tansy ragwort foliage enter a dormant stage called aestival diapause. This delays oviposition and thus is thought to be an adaptation to avoid the desiccation of eggs during the hot and dry summer (Frick and Johnson 1973). Adults resume feeding with the onset of the rainy season in fall and begin oviposition within 2 weeks. The majority of eggs hatch within 3 weeks, and larvae developing through three instars overwinter in the root crowns of tansy ragwort. At low elevations, adults are able to overwinter and lay eggs, which may hatch throughout the winter into spring. In the cooler and moister Swiss Jura Mountains, adults emerge in the summer. Oviposition begins within 2 weeks after emergence and continues throughout the summer into late fall. Few eggs may hatch in the fall following a 1–9-month facultative diapause (Frick 1971); however, field observations in Switzerland revealed that larvae emerge only the following spring (Puliafico et al. 2008) (Fig. 1). Thus, a key difference between these life cycles is that larvae of the Italian beetles are present in the fall and those of the Swiss beetles almost exclusively in the spring and that Italian beetles aestivate while Swiss beetles are active during the summer.

### Study populations

Beetles for the experiments were collected each year from one population in Idaho (Swiss ancestry) and three different populations in Oregon (Italian ancestry). The ancestry of each population was known from release records but was also confirmed by molecular analyses using amplified fragment length polymorphisms (Szűcs et al. 2011).

Swiss beetles were provided to the University of Idaho in 2004 from successful releases at high-elevation infestation sites in Montana. Beetles were reared in laboratory and greenhouse between 2004 and 2006 and in a common garden at Moscow, Idaho (780-m elevation, 46°44′N, 117°01′W), between 2007 and 2009. The laboratory rearing involved collection of eggs in July and August, which were kept at 2°C until the end of next April. Emerging larvae were transferred onto potted tansy ragwort plants and kept in a greenhouse until adult emergence. For the common garden rearing, adults were released in the summer onto caged potted tansy ragwort plants, which were embedded in mulch during the winter.

The Italian ancestry populations were sampled from one high-elevation site on Mt. Hood (1003-m elevation, 45°09′N, 121°46′W) and two low-elevation sites: Salem (84-m elevation, 44°52′N, 122°57′W) and Scherzinger road (28-m elevation, 45°07′N, 123°57′W, 3 km north of Neskowin) in 2007, 2008, and 2009. From the three Oregon sites, infested tansy ragwort plants were dug up 1–3 months prior to adult emergence and kept under identical conditions in an unlit greenhouse at 22 ± 3°C day (14 h) and 12 ± 3°C night (8 h) temperatures in Moscow, Idaho.

Adults emerging from the previously described rearings for the Swiss and Italian populations were used for all experiments. All tansy ragwort plants used for rearing and experiments were grown under standard conditions in a greenhouse from seeds collected at an infestation at Meadow Creek in Benewah County, Idaho (47°02′N, 116°44′W). The four study populations will subsequently be referred to as Mt. Hood, Salem, Scherzinger, and Swiss.

### Laboratory experiments

To assess whether the length of aestival diapause of the Mt. Hood population has evolved to be more similar to that of the Swiss than to that of the low-elevation populations in Oregon, we monitored the time between adult emergence and first oviposition in an environmental chamber over three consecutive years (2007–2009). Temperatures in the environmental chamber were kept constant throughout the season (light: 25 ± 1°C; dark: 15 ± 1°C), but the photoperiod was adjusted at the beginning of each week to follow the natural cycle as experienced in Moscow. This setting is close to what the four study populations would be exposed to in nature as it is within 2° latitude of each of the sites of origin. Moreover, it ensured that emerging beetles were not subjected to an abrupt change in photoperiod at the beginning of the experiment, the most reliable cue insects use to induce aestival diapause (Tauber et al. 1986; Danks 1987). The experiment started as soon as newly emerged beetles became available: July 13–14 each year for the Salem, Scherzinger and Swiss populations, and July 23, 2007, August 23, 2008 (following the long winter of 2007/2008), and July 27, 2009 for the Mt. Hood population. Four (2007) to five (2008 and 2009) replications per population, each consisting of five males and five females, were set up in 1.3-L volume transparent plastic cylinders. Each cylinder contained tansy ragwort foliage inserted in a piece of horticultural sponge. Eggs laid either on the tansy ragwort foliage or the horticultural sponge were counted twice a week until at least 50 eggs were found.

Given the constraints of a shorter growing season at Mt. Hood, we attempted to compare the reproductive potential of Mt. Hood beetles with that of low-elevation Oregon populations and of the Swiss population. In the 2008/2009 season, fecundity was measured on ten paired females per population, using the same environmental chamber and the previously described methods. While under natural conditions, the low-elevation Oregon populations continue with oviposition throughout the winter, most Swiss beetles stop laying eggs by around mid-October, when the first frosts occur in the Swiss Jura Mountains (U. Schaffner, personal observation). The Swiss beetles die before winter even in environmental chambers under ambient temperatures, but the Italian ancestry of Mt. Hood beetles enables them to oviposit and live through the winter under such artificial conditions. To account for the likely deaths of Mt. Hood beetles at high elevations under natural conditions due to cold temperatures, we only used egg count data until the end of October 2008 (when the first frost occurs) for this population. One female each from the Scherzinger and the Swiss populations had to be excluded from statistical analyses due to accidental death and reproductive impairment (only two deformed eggs laid), respectively.

The time constraints on development posed by shorter growing seasons at high altitudes or latitudes often lead to smaller body size in ectotherms (Abrams et al. 1996; Roff 2002; Blanckenhorn and Demont 2004). We measured the body size of 100 randomly chosen males used for the diapause experiment in 2008 to test this prediction. The beetles were of slightly different ages at the time of data collection; hence, we chose to measure males to avoid any bias that may result from the differing levels of maturity of females and thus their different egg loads that may influence the size of the abdomen. Live insects, resting on the inside of plastic vials, were measured from the frons to the tip of abdomen under a microscope to the closest 0.05 mm, using an ocular micrometer. To minimize potential effects of the rearing environment on body size, we measured fifty Mt. Hood, Salem and Scherzinger males that completed a generation at the Moscow common garden. As body size measurements on live beetles involved soft body parts that may stretch we decided to also measure body size on dead beetles along with a rigid body part, that is, the elytra. Elytra length and body sizes of 25 males of each study population were measured using deep frozen beetles collected in their home environments in 2009.

Data were analyzed using analysis of variance (anova) with ‘population’ as a fixed factor. Following anova, pair-wise comparisons of least squares means were carried out on significant effects using *t*-tests. In the anova on the length of aestival diapause, year was included as an additional fixed factor. As there were no significant population*year effects noted, the data were pooled over the 3 years for graphical presentation. Fecundity data were natural log transformed to meet the statistical assumptions of the analyses. Environmental effects on body sizes of beetles collected either in their home environment or in a common garden at Moscow were assessed by testing the effect of environment and the interaction between environment and population. The phenotypic correlation between elytra length and body size was determined using Pearson’s correlation analysis. All analyses were carried out using sas. 9.2 (SAS Institute, Cary, NC, USA).

### Field experiments

To assess local adaptation of the Mt. Hood population, we reciprocally transplanted Mt. Hood and Salem beetles between their local environments, located at 1049 and 60-m elevations, respectively. To ascertain that differentiation between populations from the low-elevation and from the high-elevation sites is because of divergent selection, rather than chance events in the population history, it would be desirable to replicate populations for each of the two habitat types (Kawecki and Ebert 2004). We therefore included the Scherzinger population as a second low-elevation population. As we know of only one high-elevation site at which *L. jacobaeae* beetles with Italian ancestry were released some 30 years ago, that is, the Mt. Hood site, we were not able to replicate populations for the high-elevation habitat type. Instead, we included the Swiss population in the experiment, which also originates from a high-elevation site but has a distinct evolutionary background.

In addition to the reciprocal transplants, a common garden experiment was set up at Moscow, Idaho (780-m elevation), which, climatically, represented an intermediate locality (Appendix S1). The three field sites have progressively shorter growing seasons, with beetle development being possible year round at Salem, during 7 months at Moscow and during 6 months at Mt. Hood (Fig. A1 in Appendix S1). To compare developmental rate and performance of the four populations, we assessed larval abundances and stages in spring and adult abundances in summer.

The same experimental designs were used for both the reciprocal transplant experiment and the common garden in Moscow and, hence, are described together. Tansy ragwort plants, to be infested with beetles, were grown in pots (3.4-L volume) from seeds beginning February 2007 in a greenhouse in Moscow. Each pot was covered with a translucent mesh cage, which enclosed both the pot and the plant, to prevent contamination by beetles from the surrounding environment, and possible larval movement between plants within the soil. Potted plants were arranged in four blocks (31–32 plants/block) and embedded in the soil at the three field sites between late April and early May in 2007. Two females and two males were released on each plant between July 12 and August 5, 2007, using a randomized complete block design to assign the four beetle populations to plants within each block (7–8 replicates/population/block). Five control plants at each location did not receive any beetles and served to test whether the covering mesh effectively excluded *L. jacobaeae* beetles from the locally occurring beetle populations.

We retrieved plants and monitored larval development and infestation rates in fall (Oct 6–7, 2007) at the Salem and the Mt. Hood sites (four replicates/population), and the next spring (June 20, 2008) at the Mt. Hood site (four Mt. Hood, six Salem, five Swiss, and two Scherzinger replicates). In the Moscow common garden, larval development and larval infestation rates were measured (eight replicates/population) in spring (May 24, 2008) and adult abundances in the last weeks of July, August, and September (8–12 replications/population). Plants retrieved from the field sites were dissected in the laboratory. Larvae found in leaf petioles, roots, and the surrounding soil were counted. The larval stage was determined by measuring the width of the head capsule of each larva to the closest 0.033 mm using an ocular ruler. Above- and belowground plant biomasses were weighed following 48 h in a drying oven at 75°C and used to assess any effect on larval abundance.

Due to damage to the cages and contamination by beetles from the surrounding area, we were not able to measure larval or adult abundances at the Salem site in spring and summer 2008. We were able to collect larval but not adult abundance data in the summer at the Mt. Hood site where spring runoff damaged most of the replications. As a result of these losses, the only data that we could directly compare in the same time period between the low-elevation (Salem) and high-elevation (Mt. Hood) sites were larval abundances in the fall. While a fall comparison is possible, the likely high winter mortality at Mt Hood makes a comparison of Salem at fall with Mt Hood at summer biologically more meaningful. Hence, we chose to compare larval abundance data as a measure of fitness at relevant periods during the season, that is, in the fall at low elevations and in the summer at high elevations.

The effect of plant biomass on mean larval numbers was assessed by means of ancova using below- and aboveground biomass as covariates for all monitoring dates and for all study sites. To assess local adaptation, anova was used on larval abundance data from the Salem site at fall and from the Mt. Hood site in summer with test site (low versus high), collection site (low versus high), and population as fixed factors. Significant interactions between test site*collection site as well as between populations within each test site*collection site combination [population (test site*collection site)] would indicate that beetles from different elevations or sites within elevation had responded differently to low and high elevation. Hence, by evaluating the direction of changes and patterns of performance, we can test the hypothesis of local adaptation. As Swiss beetles lay diapause eggs that only hatch in spring, data collected at fall for this population are not representative of their performance at low elevations. Thus, data for fall were excluded for the Swiss population from the analyses.

Differences in developmental rate or the onset of larval development were compared by examining the relative frequency of larval instar distributions among populations at the Moscow site in spring 2008 utilizing a categorical test of homogeneity. All larval and adult count data were log transformed for anova for each study site and for each monitoring date to meet assumptions of the statistical tests. In cases where data contained zero counts, 0.5 was added to all values before transformation. All data were analyzed in sas 9.2 (SAS Institute).

## Results

### Laboratory experiments

**Figure 2:**
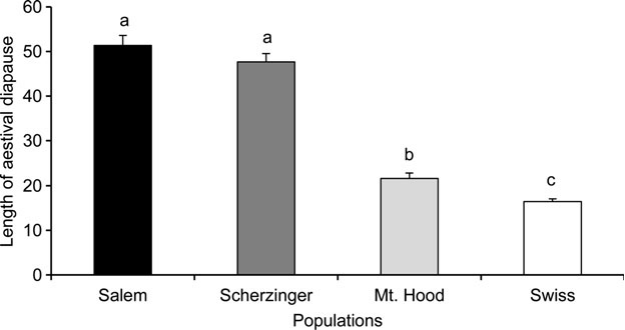
Pooled averages for 2007, 2008, and 2009 data comparing the length of aestival diapause (time between emergence and first oviposition) in days (mean + 1 SE) of four *Longitarsus jacobaeae* populations. Different letters on top of bars indicate significant difference at 95% confidence.

Aestival diapause was significantly shorter for the high-elevation populations (Mt. Hood and Swiss) than for the low-elevation populations (population: *F*_3,33_ = 172.52, *P* < 0.0001; population*year interaction: *F*_6,33_ = 1.97, *P* = 0.0992; Fig. 2). Mt. Hood females started laying eggs 3 weeks after emergence, only 5 days after the cold-adapted Swiss beetles (Fig. 2). In contrast, females from the low-elevation Salem and Scherzinger populations delayed oviposition until approximately 7 weeks after emergence.

Mt. Hood beetles laid similar numbers of eggs in autumn (until the end of October) as the Swiss beetles, and similar numbers as the Salem beetles during their entire lifetime (i.e., autumn to following spring). In contrast, Scherzinger beetles revealed a higher fecundity than the other three populations (Table 1). However, it should be noted that the lifetime fecundity of Mt. Hood beetles (without the artificial cutoff date) is similar to that of the Scherzinger beetles (data not shown).

**Table 1:** Sample sizes (*N*) and life-history parameters (mean ± 1 SE) of the four *Longitarsus jacobaeae* study populations

	Scherzinger	Salem	Swiss	Mt. Hood	*F*; *P* (trt)
*N* (females)	9	10	9	10	
Fecundity* (# of eggs)	598.2 ± 43.2^A^	281.2 ± 63.9^B^	282.2 ± 38.2^B^	357.8 ± 45^B^	5.89; 0.0024
*N*	100	100		100	
Male body size (mm)					
Home environment†	3.400 ± 0.0205^A^	3.429 ± 0.0183^A^	N/A	3.215 ± 0.0175^B^	38.17; <0.0001
*N*	50	50	100	50	
Male body size (mm)					
Moscow common garden†	3.370 ± 0.0190^B^	3.422 ± 0.0193^AB^	3.433 ± 0.0169^A^	3.271 ± 0.0229^C^	13.20; <0.0001
*N*	25	25	25	25	
Elytra size (mm)					
Home environment	2.509 ± 0.0164	2.467 ± 0.0268	2.517 ± 0.0192	2.451 ± 0.0178	2.42; 0.071

The last column gives the test statistics for anova population effects. Superscript letters indicate significant difference in populations at the 95% level of confidence for each response.

* Lifetime fecundity are shown for Scherzinger, Salem, and Swiss beetles but reproduction was cut off for Mt. Hood beetles at October 31, 2008 to mimic their normal life-cycle length in the field. Note that fecundity data were natural log transformed for statistical purposes, but the means and standard errors are shown for nontransformed values.

† Body size of males was measured using beetles reared in their local environments (home environment) or at a common garden in Moscow, Idaho. For the Swiss population, the common garden and the home environment constitute the same location.

Body sizes of males differed significantly among populations collected either from their local environments (*F*_2,297_ = 38.17, *P* < 0.0001) or from a common garden after rearing for one generation (*F*_3,246_ = 13.20, *P* < 0.0001; Table 1). Mt. Hood males were smaller in both environments than males from other populations. The rearing environment did not influence body size (environment: *F*_1,444_ = 2.09, *P* = 0.1489; population*environment interaction: *F*_2,444_ = 0.13, *P* = 0.8762). Elytra measurements revealed marginally significant differences among populations (*F*_3,96_ = 2.42, *P* = 0.071; Table 1). The correlation between elytra length and body size was significant in all populations (all *P* values < 0.0001); it was highest in the Swiss (*r* = 0.88) and lowest in the Mt Hood population (*r* = 0.72) but did not differ significantly among populations (χ^2^ = 2.66, *P* = 0.448).

### Field experiments

**Figure 3:**
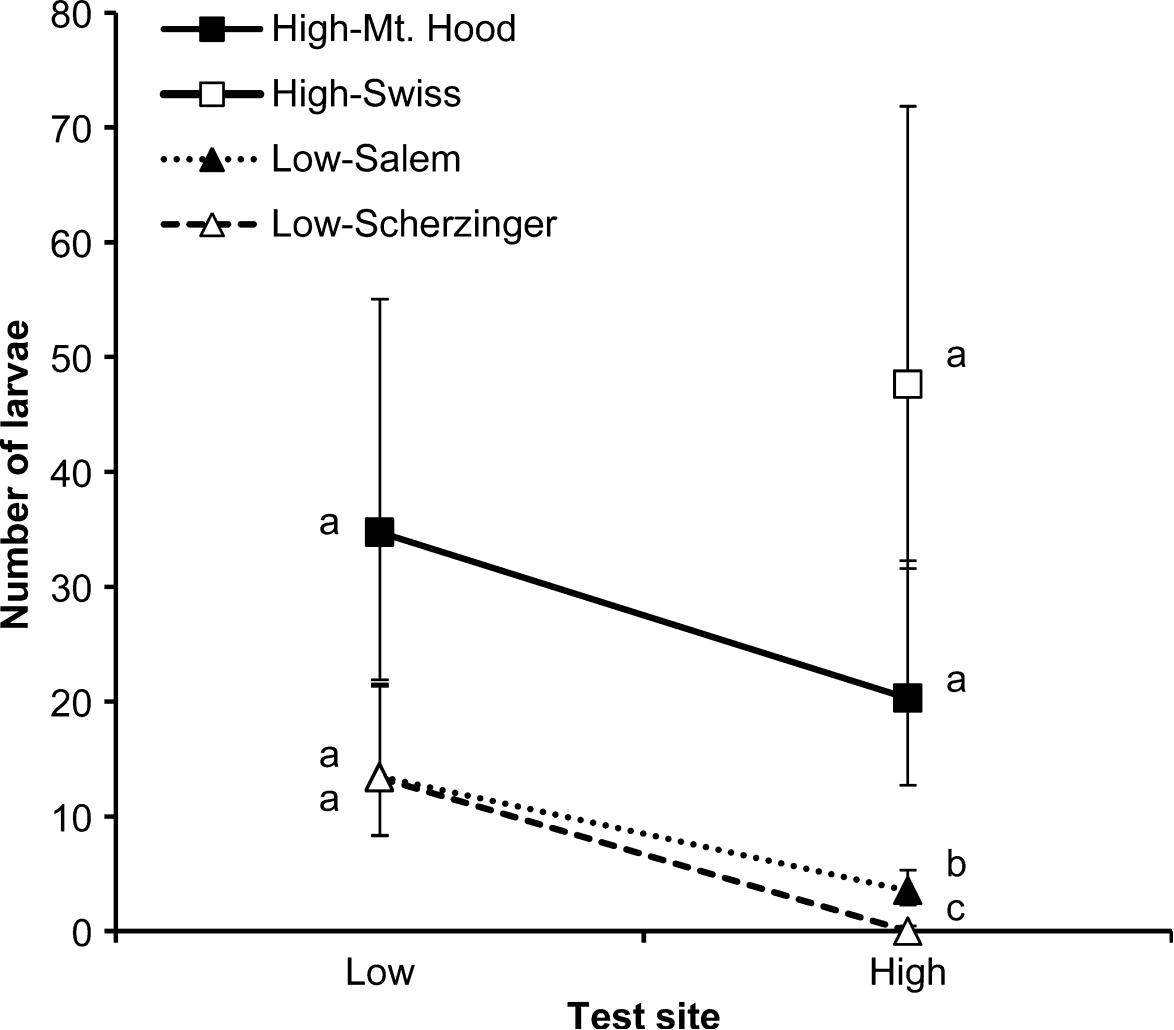
Mean number of larvae (back-transformed mean ± 1 SE) of two low-elevation (Salem, Scherzinger) and two high-elevation (Swiss, Mt. Hood) *Longitarsus jacobaeae* populations reciprocally transplanted between a low-elevation (Salem, OR) and a high-elevation (Mt. Hood, OR) test site. Means are shown for fall 2007 at the low and for summer 2008 at the high-elevation site. Data for fall were excluded for the Swiss population from the analysis (see text for details). Note that statistical tests for local adaptation were based on log-transformed data [population (test site*collection site) interaction: *F*_7,25_ = 13.01, *P* < 0.0001]. Different letters indicate significant difference at 95% confidence within each test site.

The reciprocal transplant of beetles from low-elevation (Salem and Scherzinger) and high-elevation (Swiss and Mt. Hood) populations to either low-elevation (Salem) or high-elevation (Mt. Hood) sites revealed differing performance of populations between test sites (test site*collection site interaction: *F*_6,22_ = 8.74, *P* = 0.0073; population (test site*collection site) interaction: *F*_6,22_ = 3.26, *P* = 0.0408; Fig. 3). Beetles from Salem and Scherzinger (low elevation) performed well at the low-elevation site in fall (untransformed mean larvae numbers = 23.75 and 15.5, respectively) (Fig. 3). In contrast, at the high-elevation site, these low-elevation populations had very low larval attack rates both in autumn 2007 (Appendix S3) and in the following summer (Scherzinger, untransformed mean larvae = 0.00; Salem, mean larvae = 5.00) (Fig. 3). High numbers of Swiss larvae were found in summer at the Mt. Hood site (Fig. 3). The number of larvae for the Mt. Hood population was high in the low-elevation environment and high but somewhat lower than that of the Swiss in the high-elevation environment (Fig. 3). ancova using either above- or belowground biomass as covariates revealed no significant effect of plant biomass on larval attack rate at any of the sites in fall 2007 and spring 2008 (*P* values > 0.05; data not shown).

**Figure 4:**
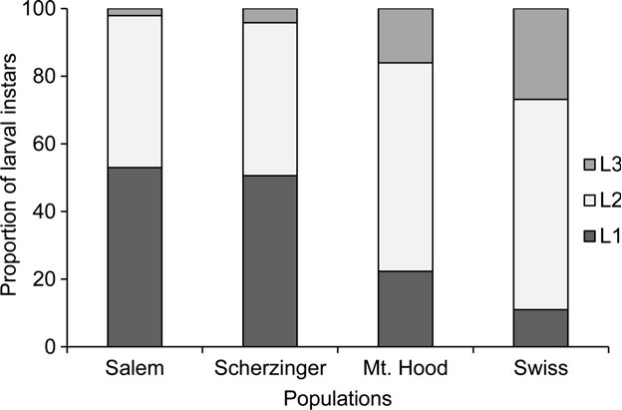
Relative frequency of three larval instars found on tansy ragwort plants infested with four *Longitarsus jacobaeae* populations at the Moscow common garden in spring 2008. Test of homogeneity of larval proportions: χ^2^ = 327.78, *P* < 0.0001.

Spring data from the Moscow site revealed significant differences in the phenology of the populations, with Swiss and Mt. Hood beetles being in more advanced stages than the low-elevation populations. At the Moscow site, the relative frequency of the three larval stages differed significantly among populations in spring (χ^2^ = 327.78, *P* < 0.0001; Fig. 4). Swiss (62% second and 27% third instars) and Mt. Hood (62% second and 16% third instar) larvae showed similar and more advanced development than the Salem and Scherzinger populations (53% and 51% first instars and 45% second instars, respectively) (Fig. 4). The number of larvae did not differ significantly among populations (*F*_3,16_ = 1.8, *P* = 0.2165) at the Moscow site (Fig. B1 in Appendix S2). There was also no difference among populations in the number of adults emerging during the summer of 2008 (*F*_3,25_ = 0.75, *P* = 0.55; Fig. B2).

## Discussion

Our results suggest that Italian ancestry beetles from lowland Oregon introduced to the high-elevation environment of Mt. Hood underwent rapid evolution in <30 generations. The observed life-history changes in the Mt. Hood population are in accordance with results from theoretical (Abrams et al. 1996; Roff 2002; Bradshaw and Holzapfel 2008) and empirical studies (e.g., Dingle et al. 1990; Blanckenhorn and Fairbairn 1995; Orr 1996; Berner et al. 2004), indicating that insects respond to shorter-growing seasons by accelerating their development. The evolved lack of aestival diapause enables Mt. Hood beetles to oviposit earlier and lay similar number of eggs in shorter amount of time than Salem beetles during their entire lifetime. The phenological shifts in larval development of the Mt. Hood population observed in the Moscow common garden experiment are either due to earlier onset of larval development or to accelerated larval development. These trait changes resulted in a Mt. Hood population, whose phenology now more closely resembles that of the cold-adapted Swiss population than that of the low-elevation populations it originated from. However, there are still marked differences between the Mt. Hood and Swiss phenologies, such as the emergence of larvae in fall for the Mt. Hood population, which may be maladaptive in the high-elevation environment as larvae may have higher mortality than eggs during the harsh winters. Abrams et al. (1996) hypothesized that accelerating the development in response to shorter growing seasons may lead to smaller adult size. Indeed, male body size of Mt. Hood beetles is smaller than that of the low-elevation beetles. In contrast, elytra length differs only marginally among the populations, suggesting that its regulation may be partially independent from that of body length (Shingleton et al. 2007). Accordingly, the correlation coefficient between body size and elytra length is lowest in the Mt Hood population, but it does not differ significantly from those of the ancestral Italian populations. Interestingly, the cold-adapted Swiss beetles tend to be larger than Italian ancestry beetles. According to Blanckenhorn and Demont (2004), ectotherms are equally likely to become smaller (converse Bergmann cline) or larger (Bergmann cline) in colder environments. The different responses can be explained by different proximate mechanisms; Bergmann clines are mediated by temperature, while converse Bergmann clines are an adaptive response to time constraints by shorter growing seasons (Blanckenhorn and Demont 2004).

The correspondence between predictions and the observed life-history changes in the Mt. Hood population suggest that the trait changes are adaptive. The reciprocal transplant experiment seems to confirm that. However, the results should be interpreted with caution as we were not able to directly compare fitness of populations in terms of larval and/or adult abundances in the summer 2008. The loss of the Salem site following the fall 2007 monitoring did not allow this comparison. Thus, comparisons were based on larval abundance in autumn at the low-elevation site and in the following summer at the Mt. Hood site. Most importantly, we found better performance of the Mt. Hood population in its home environment compared with the Salem and Scherzinger populations. The number of plants dissected in spring 2008 was relatively low. Nevertheless, the number of larvae found at the Mt. Hood site was six-fold higher for the Mt. Hood (on average 29 larvae/plant) compared with the lowland Salem and Scherzinger populations (Fig. 3). The findings may reflect lower egg or larval survival of the lowland populations relative to the Mt. Hood population during the winter. Increased larval survival of the Mt. Hood population might be a consequence of earlier larval hatching in fall and more advanced and/or heavier overwintering larvae. In any case, the results indicate that Mt. Hood beetles have become adapted to this environment or are in the process of adaptation as they do not perform quite as well as Swiss beetles (Fig. 3).

Adaptation to the high-elevation environment apparently did not result in reduced performance of Mt. Hood beetles at low elevation (Fig. 3). In fact, Mt. Hood seemed to perform better in the low-elevation environment than the local populations. One explanation might be higher fitness of the Mt. Hood population than of the two lowland populations in this environment. This might be a plausible scenario given findings of a review on local adaptation, which concluded that adaptation is often not costly, and that one population can have superior fitness in multiple environments without trade-offs (Hereford 2009). However, if the fitness of Mt. Hood is higher at the low- as well as in the high-elevation environment than those of Salem and Scherzinger, it should have also performed best at the intermediate elevation in Moscow, which was not the case (Figs B1, B2 in Appendix S2). Hence, a more likely alternative explanation for the higher larval densities of the Mt. Hood population detected in fall at low elevation is that larval emergence of the lowland populations was delayed due to their later oviposition relative to the Mt. Hood population. The relatively early monitoring date in fall, which was chosen based on the level of ongoing damage to the cages, likely did not capture a significant proportion of emerging larvae of the Salem and Scherzinger populations.

We cannot fully exclude the potential influence of maternal effects on our results, because most beetles used in our experiments emerged from field-collected juveniles. However, our rearing method, which involved the collection of beetles when they were still in early to late larval stages and rearing them under common conditions, should have minimized maternal effects. Maternal effects in insects are most prevalent in the early stages of development and are expressed most commonly between mother and embryo (Danks 1987; Mousseau and Dingle 1991). Moreover, Reinhold (2002) found that large (detectable) maternal effects on life-history traits (e.g., development time, diapause response, longevity, oviposition pattern) were rare in insects. The fact that offspring of Italian ancestral and Swiss beetles resulted in offspring with intermediate diapause response (Frick and Johnson 1973; Szűcs et al. 2012) indicates that the time between adult emergence and first oviposition is at least partly genetically determined in *L. jacobaeae*.

Our results suggest that contemporary evolution in *L. jacobaeae* has influenced the outcome of the classical biological control project against *J. vulgaris* by reducing tansy ragwort also at a high-elevation site (McEvoy et al. 1991). So far, experimental evidence is sparse for post-introduction evolution in biological control agents (Hufbauer 2002; Phillips et al. 2008; Bean et al. 2012; McEvoy et al. 2012). We propose that the prevalence and relevance of contemporary evolution in biological control systems has been underestimated and understudied so far, considering the number of biological agents exposed to novel environmental conditions. Establishment of biological control agents in colder climates (i.e., in Canada) than their area of origin (Myers and Sabath 1980; Murray 1982; Harris 1984) suggests that climatic adaptations may have happened in other agents as well. The lack of evidence for contemporary evolution in biological control systems reflects a general historical perception that contemporary evolution is relatively rare. However, once biologists started to look for contemporary evolution in the wild, they found evidence for it more often than expected (Stockwell et al. 2003).

If evolution is common post-introduction, it has implications for strategies for biological control agent collections in the native range and redistributions within the exotic range. For example, the utility of the currently widely used practice of climate matching between the collection and release area of agents should be reevaluated in light of the likelihood of post-introduction climatic adaptations. Similarly, nontarget species that are accepted for oviposition and suitable for larval development, that is, that are within the fundamental host range of a biological control agent, may have to be considered to experience some level of nontarget effects even if they occur outside the climatic niche occupied by the biological control agent in the native range.

Biological control release practices should also utilize evolutionary theory and conduct releases and redistributions in ways that maximize the establishment success and the adaptive potential of populations. For example, when nonadapted organisms are released into a new environment the initial high mortality will likely cause large fluctuations in population size. Releases at this point should continue in subsequent years to provide both genetic rescue by reducing inbreeding, increasing genetic variation and thus the adaptive potential of the released populations (Tallmon et al. 2004; Hedrick et al. 2011) and demographic rescue by sustaining the population for long enough that adaptation can occur (Holt and Gomulkiewicz 1997). However, once establishment in the new environment is confirmed gene flow from nonadapted populations should be ceased or kept at a relatively low level as high levels of gene flow likely compromise adaptation (Gomulkiewicz et al. 1999; Boulding and Hay 2001; Garant et al. 2007; Hendry et al. 2011).

Empirical data from biological invasions provided some support that contemporary evolution is more common than historically thought and also provided insight into the factors that may hinder or promote contemporary evolution (e.g., Maron et al. 2004; Dlugosch and Parker 2008; Colautti et al. 2010). However, there is still much to be learned to better understand the mechanisms and their interactions that shape responses of populations facing environmental change. While the assessment of contemporary evolution in invasive species is limited by the fact that the exact sources of introductions are rarely known with certainty (Dlugosch and Parker 2008; but see Handley et al. 2011 and references therein), biological control projects offer a great opportunity to test rapid evolution as the timing, origin, and the scale of introductions are usually well documented. Hence, evolving populations in the exotic range can be compared directly with their population of origin and the time frame within which the changes take place can easily be assessed. We therefore advocate for designing ongoing and new releases or redistributions in biological control programs in an explicitly experimental way to test not only ecological, but also evolutionary hypotheses. This would advance our general understanding of the interface between evolutionary and ecological processes and ultimately also lead to improved establishment of control agents and increased biological control success rates.

## Data archiving statement

Data used to generate the main results of this paper are available as Supporting Information on the Evolutionary Applications Web site.

## Supporting Information

**Data S1.** Data.

**Appendix S1.** Characteristics of the common garden locations.

**Appendix S2.** Results of spring and summer monitoring at the Moscow, Idaho common garden in 2008.

**Appendix S3.** Results of monitoring at the high elevation site (Mt. Hood, Oregon) in fall 2007.
