# Supplementary material for: Post-introduction evolution in the biological control agent Longitarsus jacobaeae (Coleoptera: Chrysomelidae)
Source: Evol Appl. 2012 Dec;5(8):858–68. doi: 10.1111/j.1752-4571.2012.00264.x (PMC3552403; doi:10.1111/j.1752-4571.2012.00264.x)
Supplement: Supplementary file 1 [file eva0005-0858-SD1.pdf]

Appendix A: Characteristics of the common garden locations

The Mt. Hood site was located (45°10' N, 121°46' W) on a clearing surrounded by spruce, hemlock and lodgepole pine dominated forest. The Salem site was situated along a strip of grassland next to a small lake at the edge of town at the Oregon State Department of Agriculture Research Station (44°55' N, 122°59' W). The Moscow site was located on old farmland at the University of Idaho's H.C. Manis Entomological Research Laboratory next to a wheat field.

The length of the growing season at each site is estimated from growing degree days calculated at 10°C base temperature (WRCC 1951-2010), which is probably a conservative estimate for Italian beetles. No development was observed for another flea beetle, *Apthona abdominalis* from Italy at 12 °C (Fornasari 1995), and literature review estimated the average lower developmental threshold for Coleoptera as 10.6-12.1°C (Honek and Kocourek 1990). Growing degree days at 10°C base temperature decrease from 1218 at Salem, through 1029 at Moscow, to 460 at Mt. Hood (WRCC 1951-2010). Mean summer temperatures are similar for the Moscow and Salem sites, and approximately 5°C lower at the Mt. Hood site (WRCC 1971-2000). Mean winter temperatures are about 5-6°C lower at Mt. Hood and Moscow than at Salem. This difference was even more pronounced in the 2007/2008 growing season when mean temperatures at Mt. Hood were up to 3°C below average almost year round (NCDC 2007, 2008). Mean monthly temperatures close to Rome, Italy (NCDC 1955-1980) from where Italian beetles were introduced most closely resemble the conditions encountered at Salem, while climate experienced by Swiss beetles (MeteoSwiss 1961-1990) is very similar to that of Mt. Hood (Fig. S1).Climatological data from the nearest National Weather Service meteorological station were used for U. S. sites (WRCC 1971-2000), the Vigna di Valle weather station 36 km from Rome for Italy (NCDC 1955-1980), and the La Chaux de Fonds weather station 15 km from Saint Imier for Switzerland (MeteoSwiss 1961-1990).

References

Fornasari, L. 1995. Temperature effects on the embryonic development of *Aphthona abdominalis* (Coleoptera: Chrysomelidae), a natural enemy of *Euphorbia esula* (Euphorbiales: Euphorbiaceae). Environ. Entomol 24:720-723.

Honek, A., and F. Kocourek. 1990. Temperature and development time in insects: a general relationship between thermal constants. Zoologische Jahrbücher, Abteilung für Systematik, Ökologie und Geographie der Tiere 117:401-439.

MeteoSwiss, Federal office of Meteorology and Climatology. Swiss Confederation. http://www.meteoswiss.admin.ch/web/en/climate/swiss_climate/tabellen.html

NCDC, National Climatic Data Center US Department of Commerce. http://www.ncdc.noaa.gov

WRCC, Western Regional Climate Center. http://www.wrcc.dri.edu

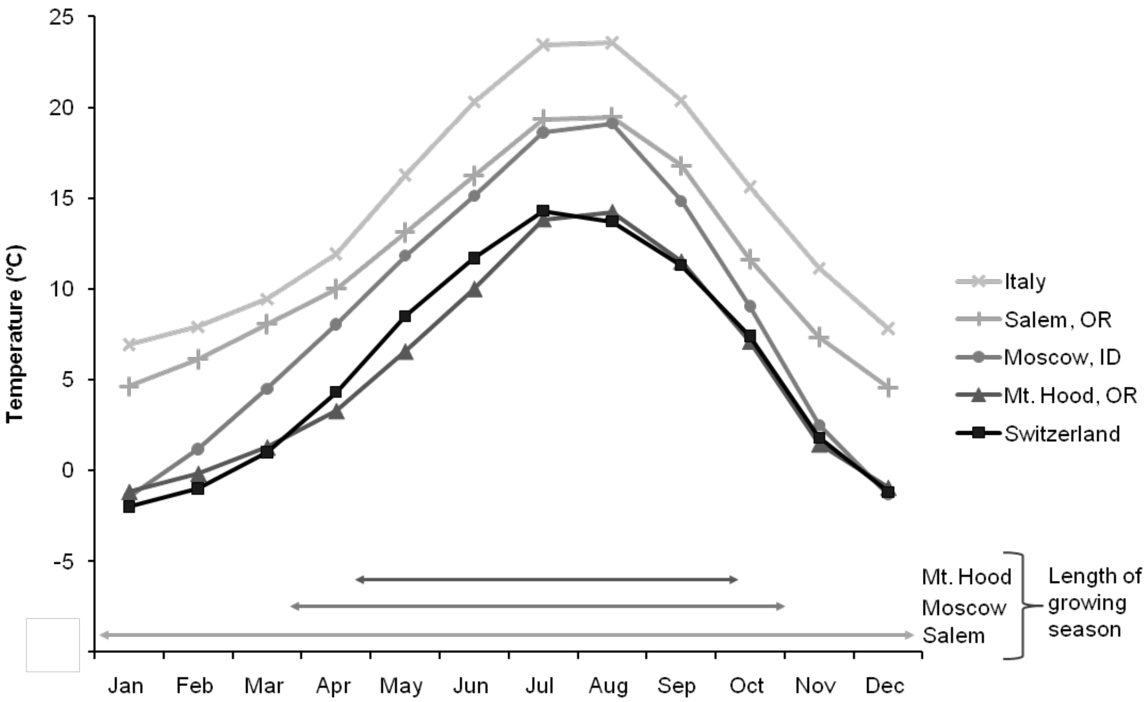

Figure A1. Mean monthly temperatures for the three common garden locations (Salem, OR; Moscow, ID; and Mt. Hood, OR), and for two sites in Italy and Switzerland, from which *Longitarsus jacobaeae* was introduced to the U.S. Lengths of growing seasons are approximated using growing degree days calculated at 10°C base temperature.

Appendix B: Results of spring and summer monitoring at the Moscow, Idaho common garden in 2008

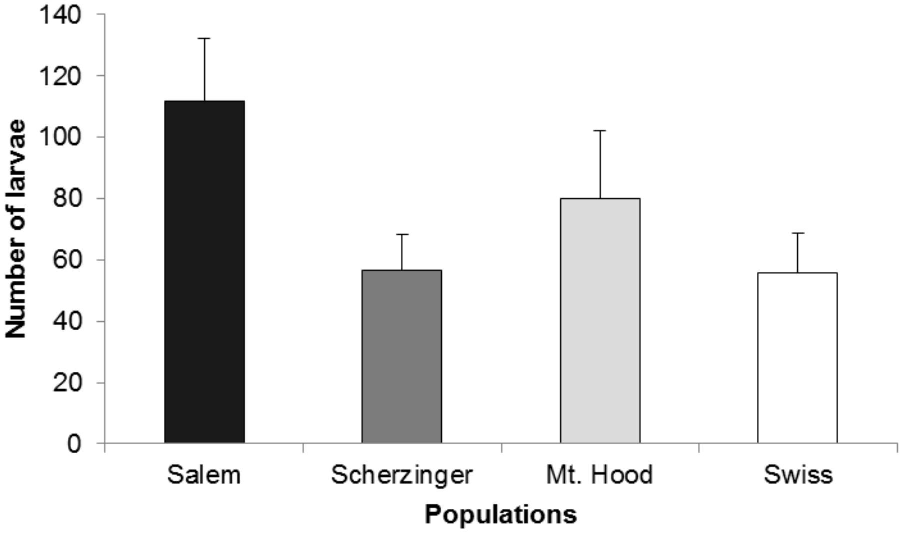

\

Figure B1. Mean number of larvae (mean ± SE) of four *L. jacobaeae* populations at the Moscow common garden in spring 2008. Note that statistical tests were based on log-transformed data (block: $F_{3,16} = 0.22$, $P = 0.8845$; population: $F_{3,16} = 1.8$, $P = 0.2165$).

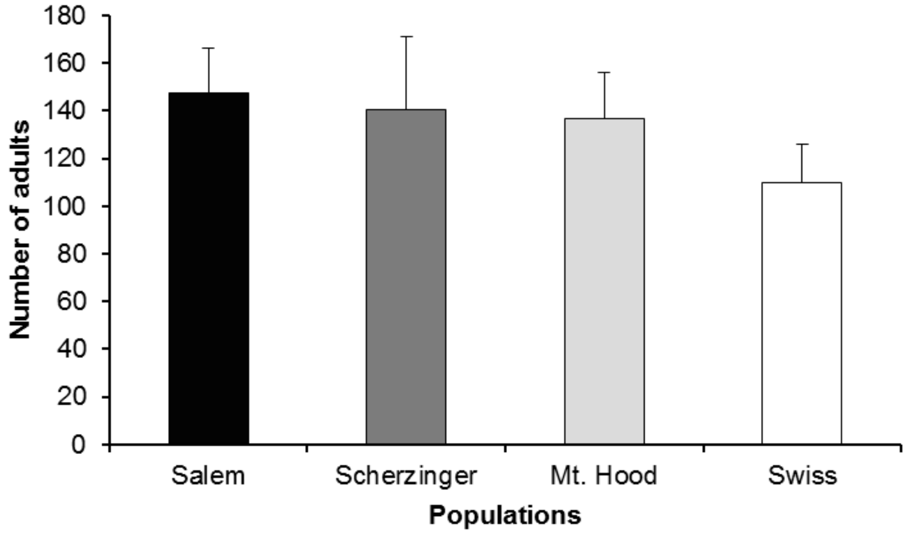

Figure B2. Mean number of adults (mean ± SE) of four *L. jacobaeae* populations emerging at the Moscow common garden in summer 2008. Note that statistical tests were based on log-transformed data (block: $F_{3,25} = 0.27$, $P = 0.8497$; population: $F_{3,25} = 0.75$, $P = 0.55$).

Appendix C: Results of monitoring at the high elevation site (Mt. Hood, Oregon) in fall 2007

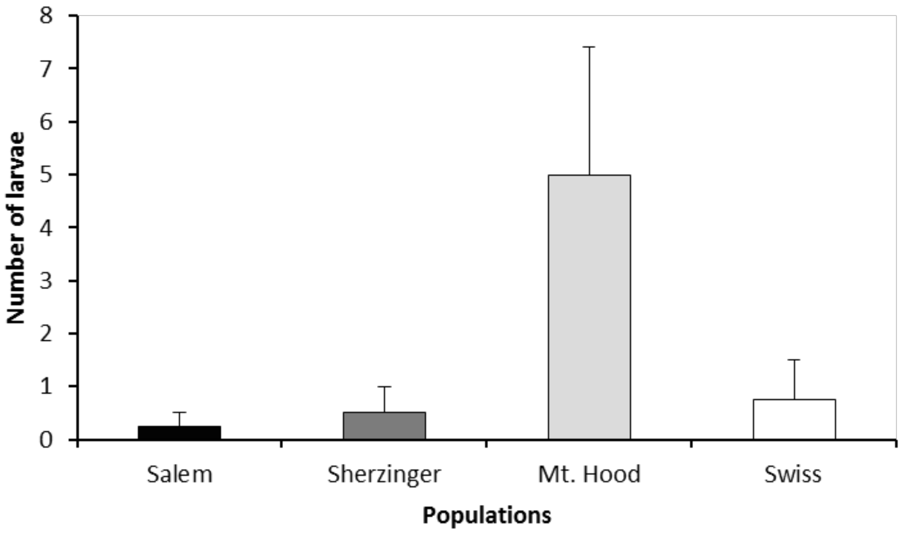

\

Figure C1. Mean number of larvae (mean ± SE) of two low elevation (Salem, Scherzinger) and two high elevation (Swiss, Mt. Hood) *L. jacobaeae* populations at the high elevation (Mt. Hood, OR) test site in fall 2007. Note that statistical tests were based on log-transformed data (block: $F_{3,9} = 0.11$, $P = 0.9519$; population: $F_{3,9} = 3.53$, $P = 0.0618$).
